# Non-Psychoactive Cannabis Extract Disrupts Reinstatement and Reconsolidation in Cocaine-Induced Conditioned Place Preference in Mice

**DOI:** 10.3390/brainsci16060585

**Published:** 2026-05-29

**Authors:** Fabián Leonardo Barreto, María Constanza Lozano, Yoshie Adriana Hata, Aura Rocio Hernández, Jorge A. Martínez-Ramírez

**Affiliations:** 1 Department of Pharmacy, National University of Colombia, Bogotá 14490, Colombia;; 2 Department of Biomedical Science, Faculty of Health and Society, Malmö University, 205 06 Malmö, Sweden; 3 Biofilms Research Center for Biointerfaces, Malmö University, 205 06 Malmö, Sweden

**Keywords:** non-psychoactive cannabis extract, cocaine, conditioned place preference, reconsolidation, reinstatement

## Abstract

**Highlights:**

**What are the main findings?**

• Non-psychoactive cannabis extract inhibits both priming- and stress-induced reinstatement of cocaine-seeking behavior in mice.
• Non-psychoactive cannabis extract showed consistency with disruption of reconsolidation-like processes of cocaine-associated memory traces.

**What are the implications of the main findings?**

• Suggests that NPCE may modulate relapse-like behavior across multiple triggers in a preclinical CPP model, supporting further investigation in cocaine use disorder research.
• Indicates that targeting memory reconsolidation-like processes may contribute to a reduction in the persistence of drug-associated memories in preclinical models.

**Abstract:**

Background: Cocaine use disorder (CUD) remains a major global health concern, with no FDA-approved pharmacological treatments currently available. Cannabidiol (CBD), a non-psychoactive phytocannabinoid derived from *Cannabis sativa* L., has shown promising preclinical effects in disrupting the consolidation and retrieval of drug-associated memories, thereby attenuating relapse-like behaviors. Objectives: The present study evaluated the effects of a low-THC CBD-rich cannabis extract (NPCE) on the reinstatement and reconsolidation of cocaine-induced conditioned place preference (CPP) in male CD1 (ICR) mice, an approach not previously investigated. Methods: The extract was administered at a dose equivalent to 20 mg/kg of CBD. Treatment significantly attenuated both priming- and stress-induced reinstatement of cocaine-induced CPP. Reinstatement was triggered either by a cocaine priming injection or by acute stress exposure, whereas reconsolidation-like processes were assessed by administering the extract following memory reactivation sessions and subsequently evaluating the persistence of cocaine-associated preference over time. Results: NPCE showed a consistent result with disruption of reconsolidation-like processes of cocaine-associated memory, with effects persisting for at least two weeks. The extract alone did not induce conditioned preference or aversion. Conclusions: These findings suggest that NPCE modulates drug-associated memory processes involved in relapse-like behavior. However, the underlying mechanisms were not directly evaluated and remain to be elucidated. Further studies are warranted to include both sexes, evaluate effects across multiple behavioral paradigms, directly compare full-spectrum extracts with isolated cannabinoids, and incorporate receptor-specific approaches to clarify the mechanisms of action.

## 1. Introduction

Cocaine, a tropane alkaloid extracted from *Erythroxylum coca*, is widely used as a recreational drug. In illegal markets, it is primarily available in two forms: cocaine base (commonly smoked) and cocaine hydrochloride in powder form, typically administered via nasal insufflation or intravenous injection [1]. According to the 2024 World Drug Report by the United Nations Office on Drugs and Crime (UNODC), an estimated 23 million people used cocaine in 2022. This number reflects a significant increase in global cocaine production and consumption compared with previous reports, contributing to an increase in associated health problems and drug-related violence [2]. Despite the growing number of individuals with cocaine use disorder (CUD) worldwide, the U.S. Food and Drug Administration (FDA) has not approved any pharmacological treatment [1].

Cannabidiol (CBD), a major non-psychoactive cannabinoid found in the *Cannabis sativa* L. plant, has been the subject of numerous studies due to its potential therapeutic properties in the treatment of substance use disorder (SUD). CBD lacks intrinsic rewarding or hedonic properties [3], and preclinical and clinical studies have explored its impact on the consumption of various drugs, including psychostimulants, alcohol, opioids, nicotine, and cannabis [4]. As a potential treatment for cocaine addiction, CBD has shown beneficial effects across multiple domains, including modulation of cocaine-related reward, reduction in cocaine consumption, anxiolytic effects, promotion of neurogenesis, and hepatoprotective properties [5]. In preclinical studies, CBD has been evaluated in the conditioned place preference (CPP) paradigm, including cocaine-induced CPP [6,7,8,9,10]. This paradigm is a widely used Pavlovian learning model designed to evaluate the motivational properties of substances and the memories associated with their use [11]. The CPP procedure comprises three main phases: preconditioning (Pre-C), where the animal’s baseline preference is determined; conditioning (Cond), in which the substance is paired with one chamber and the vehicle with the other; and the test phase, during which, in a drug-free state, the time spent in each chamber is recorded. Following the conditioning phase, the persistence of CPP memory and relapse-like behavior can be evaluated through extinction and reinstatement procedures. These approaches are commonly employed to model the persistence of drug-associated memories and relapse. Alternatively, reconsolidation procedures can be employed, whereby previously consolidated memories become transiently labile upon reactivation, allowing their modification or attenuation [12].

CBD can disrupt the reconsolidation of cocaine-induced CPP memories, thereby reducing the risk of relapse [9]. Ledesma et al. [10] found that CBD not only prevented priming-induced reinstatement of cocaine CPP, but also reduced locomotor stimulation and mitigated memory deficits associated with cocaine withdrawal in mice. Nevertheless, some studies have found that CBD does not reduce cocaine self-administration or cue-induced cocaine seeking, suggesting inconsistent behavioral outcomes [13]. CBD has been reported to suppress cocaine-induced CPP in rodents with a U-shaped dose–response profile, affecting neuronal activation in the prelimbic cortex. According to the authors, intermediate doses of CBD (20–30 mg/kg) are more effective at modulating behavioral responses in cocaine-induced CPP than high doses (120 mg/kg) or low doses (10 mg/kg) [14]. Doses of 10 mg/kg or less are ineffective in treating the acquisition of cocaine self-administration, extinction, or reconsolidation of cocaine-induced CPP, but can produce significant effects on acquisition [8,13]. In contrast, doses of 20–30 mg/kg produce significant effects on indicators of reinstatement and reconsolidation of cocaine-induced CPP [10,15], but have limited effects on abstinence-related behaviors and cocaine craving [16].

Although numerous preclinical studies have demonstrated beneficial effects of CBD on cocaine addiction, clinical evidence suggests that CBD may produce mixed outcomes [17,18,19]. For instance, in a double-blind study conducted by Meneses-Gaya et al. [17], CBD was found to be ineffective in treating withdrawal symptoms associated with crack cocaine addiction. Similarly, Mongeau-Pérusse et al. [18] reported that CBD did not produce significant effects on CUD-related outcomes in a placebo-controlled trial. Moreover, recent findings indicate that even relatively high doses of CBD (800 mg) fail to improve cognitive performance in individuals diagnosed with CUD, suggesting limited efficacy in modulating CUD-related symptoms [19]. In contrast, whole-plant cannabis and its constituent compounds have been proposed as potential therapeutic approaches for CUD in humans. Emerging evidence suggests that cannabis use may modulate both the pharmacokinetics and subjective effects of cocaine. For example, Murray et al. [20] investigated the pharmacokinetic interactions between cannabis and cocaine, reporting that cannabis use reduced plasma concentrations of cocaine and improved subjective measures associated with smoked cocaine use. Furthermore, observational studies indicate that the intentional use of cannabis as part of harm-reduction strategies may alleviate several symptoms associated with crack cocaine dependence, including sleep disturbances, paranoia, legal complications, and craving [21]. Consistently, Labigalini et al. [22] also reported beneficial outcomes associated with cannabis use, with 68% of participants indicating improvements in addiction management, including reduced craving and facilitation of abstinence from crack cocaine.

Taken together, these findings suggest that the therapeutic effects observed with cannabis-based interventions may not be attributable to a single compound, but rather to the combined action of multiple phytochemicals. The interactions among the various constituents of cannabis involve multiple pharmacokinetic and pharmacodynamic mechanisms, including the competitive inhibition of cannabinoid metabolism by cytochrome P450 enzymes and carboxylesterases [23], modulation of cannabinoid and vanilloid receptor activity [24], and interactions between cannabinoid receptor type 2 (CB2) and cannabis-derived terpenes [25]. These potential synergistic interactions have demonstrated therapeutic potential in disorders such as cancer [26] and epilepsy [27]; however, they remain insufficiently characterized in the context of substance use disorders.

Importantly, this framework provides a mechanistic basis to hypothesize that cannabis-derived formulations containing multiple constituents may produce behavioral effects that differ from those observed with CBD alone, particularly in domains related to reward processing, memory reconsolidation, and relapse.

Consistent with this framework, preclinical evidence suggests that the combined administration of CBD and Δ9-tetrahydrocannabinol (THC) facilitates the extinction of cocaine-induced CPP, although it appears to have no effect on the context-dependent locomotor sensitization to cocaine [28]. Moreover in a previous preclinical study conducted by our research group, NPCE (defined according to Colombian regulations as containing less than 1% THC on a weight/weight basis) was evaluated in a murine model of smoked cocaine exposure and showed promising effects in reducing behavioral indicators associated with cocaine consumption [29]. In the present study, the term ‘non-psychoactive cannabis extract’ is used in a regulatory and compositional sense to describe a low-THC CBD-rich cannabis extract, and not as a direct demonstration of absent psychoactive or neuropharmacological effects. To our knowledge, no previous studies have systematically evaluated the effects of a low-THC CBD-rich cannabis extract full-spectrum on both reinstatement and memory reconsolidation processes in cocaine-induced CPP. Based on this rationale, the present study aimed to evaluate the effects of NPCE on priming- and stress-induced reinstatement, as well as on the reconsolidation of cocaine-induced CPP. Additionally, the potential reinforcing or aversive properties of NPCE were assessed using an NPCE-induced CPP paradigm, to determine its intrinsic motivational effects. While these findings are consistent with the potential contribution of multiple cannabis constituents, the present study evaluates NPCE as a single pharmacological entity. Accordingly, the experimental design does not allow for the dissection of individual or synergistic contributions but rather aims to characterize the overall behavioral effects of this formulation. We hypothesized that: (i) NPCE would not produce conditioned preference or aversion on its own, indicating a lack of intrinsic reinforcing or aversive effects; (ii) NPCE would attenuate both priming- and stress-induced reinstatement of cocaine-induced CPP; and (iii) NPCE would disrupt the reconsolidation of cocaine-associated memories.

## 2. Materials and Methods

### 2.1. Animals

The study was approved by the Ethics Committee of the Faculty of Sciences at the Universidad Nacional de Colombia (Approval No. Acta 06-2023). All experimental procedures were conducted in accordance with institutional ethical standards, ARRIVE reporting guidelines [30], and internationally accepted principles for laboratory animal care. A total of 63 male CD1 (ICR) mice, aged 8 to 10 weeks, weighing 35–40 g, were housed in groups in standard stainless-steel cages (30 cm × 20 cm × 15 cm) under controlled environmental conditions. Cage positions within the animal facility were periodically rotated to avoid location-related biases. All experiments were conducted at the same time of day to control for circadian influences. Temperature was maintained at 22 ± 2 °C with a relative humidity of 60–65%. The mice were kept on a 12-h light/dark cycle, with water and food provided *ad libitum* (LabDiet standard rodent chow).

Animals were monitored daily for general health and welfare, including body weight, grooming behavior, posture, and locomotor activity. Humane endpoints were predefined and included criteria such as significant weight loss (>20% of initial body weight), persistent hypoactivity, signs of distress (e.g., piloerection, hunched posture), or any condition compromising animal welfare, in which case animals would be removed from the study and, if necessary, humanely euthanized. No unexpected adverse effects were observed during the study. Environmental enrichment was not provided in order to maintain standardized housing conditions throughout the behavioral procedures, thereby minimizing potential variability in reward-related learning and CPP responses [11]. For this study, animals were randomly assigned to the six experimental groups after completion of the pre-conditioning (Pre-C) and conditioning (Cond) phases using the randomization function implemented in AnyMaze^®^ software version 7.15 (Stoelting Co., Wood Dale, IL, USA). This procedure ensured balanced allocation based on baseline chamber preferences and conditioning performance, as described in Table 1. Allocation was not concealed, as group assignments were known during treatment administration and data analysis. Behavioral data acquisition and analysis were performed using an automated tracking system (AnyMaze^®^), thereby minimizing experimenter-related bias. The primary outcome measure (time spent in the drug-paired compartment) was automatically quantified using predefined software parameters.

All behavioral experiments were conducted during the light phase of the cycle. No genetic modifications were present, and animals were not genotyped, as CD-1 is an outbred strain. All animals were experimentally naïve and had not undergone prior experimental procedures.

Sample size was determined based on previous studies employing similar CPP paradigms and cannabinoid interventions (e.g., [10]), as well as on effect sizes commonly reported in behavioral neuroscience. No formal a priori power analysis was conducted for this study. Under these conditions, group sizes of *n* = 10 animals are typically sufficient to detect biologically relevant differences with adequate statistical power (α = 0.05), while adhering to ethical principles for animal use. Effect sizes for the primary outcomes are reported in the Results section.

### 2.2. Chemicals and NPCE

Reference standards of cocaine (1 mg/mL solution in methanol) and a cannabinoid mixture (1 mg/mL solution in methanol of mixed CBD, THC and CBN) were obtained from Merck (Darmstadt, Germany). These standards were used for the quantification of cannabinoids in NPCE, the determination of cocaine purity, and the quality control of injectable preparations.

NPCE was obtained by supercritical fluid extraction and provided by Medcolcana Organics (Bogotá, Colombia). NPCE is a multi-component botanical formulation, characterized by a CBD content of 41.05% (*w*/*w*), along with minor cannabinoids including Δ9-tetrahydrocannabinol (THC, 0.70%), cannabinol (CBN, 0.61%), cannabigerol (CBG, 0.24%), cannabichromene (CBC, 0.04%). In addition, the extract contains several terpenes, the most abundant of which were α-pinene (0.037%), α-terpinolene (0.019%), caryophyllene (0.248%), α-humulene (0.023%), α-bisabolol (0.077%), aromandendrene (0.047%), zingiberene (0.027%), and cis-3-hexenyl benzoate (0.017%) (Supplementary Material S1). Cannabinoid and terpene composition was determined by GC-MS and GC-MS/HS/SPME. Cocaine (97.5%), used as the reinforcing drug, was provided by the Anti-Narcotics Directorate of the National Police (DIRAN). All working solutions of NPCE and cocaine were freshly prepared on the day of each experiment prior to administration.

### 2.3. Doses

The cocaine dose (15 mg/kg) was selected according to Farrell et al. [31], and was prepared daily in 0.9% NaCl saline solution (SAL). The dose of NPCE, standardized according to its CBD content dose of 20 mg/kg, was determined based on previous studies by Nedelescu et al. [14] and Galaj et al. [32]. Based on its composition, the administered NPCE dose corresponds to an approximate total extract dose of 48.7 mg/kg required to achieve the target CBD content. NPCE was dissolved in a vehicle solution (VEH) consisting of SAL and 2% Tween 80 (polysorbate 80). All substances were administered intraperitoneally (i.p.) in a volume of 0.01 mL/g to the experimental animals. Previous studies have reported that Tween 80 does not significantly affect cocaine-induced CPP behavior, supporting its use as a vehicle in this paradigm [33].

### 2.4. Experiments

A conditioned place preference box (CPPb; 90 × 25 × 20 cm) was used in this study. The apparatus consisted of two separate chambers with distinct visual (a gray chamber and a blue chamber), tactile, and olfactory cues connected by a neutral zone, thereby facilitating contextual discrimination during conditioning procedures, as previously described [34].

All animals (N = 63) underwent a conditioning (Cond) phase using a biased CPP protocol, in which cocaine was paired with the initially non-preferred compartment during the Pre-C phase [11]. This approach is commonly employed to control for baseline side preferences and to improve detection of drug-induced conditioning effects. In biased CPP protocols, conditioned preference is more consistently observed when the drug is paired with the initially non-preferred compartment.

Behavioral data were acquired and analyzed using automated tracking software, enabling objective quantification of animal position and movement throughout the CPPb. The primary outcome measure was the time spent in each chamber, expressed as the difference between the drug-paired and vehicle-paired compartments, as an index of conditioned preference. Locomotor activity was not prospectively collected or analyzed as an independent outcome measure, as the study was primarily designed to evaluate motivational and associative processes related to conditioned place preference.

• Pre-conditioning (Pre-C phase)

On day 1, animals were allowed to explore the CPPb freely for 15 min, with unrestricted access to both chambers. Animals spending ≥65% of the total exploration time in either chamber were excluded, as previously described in CPP paradigms. On days 2 and 3, all animals remained in their home cage.

• Conditioning phase (Cond phase)

From days 4 to 7, animals received VEH (i.p.) in the morning and were placed in their initially preferred chamber for 20 min. Six hours later, animals were administered cocaine (15 mg/kg, i.p.) and confined to the initially non-preferred chamber for 20 min.

On day 8, a conditioned preference test was conducted, allowing all animals to freely explore the CPPb for 15 min. As an a priori individual exclusion criterion, animals were required to spend at least 1 min more in the conditioned chamber compared to the unconditioned (VEH) chamber during the Cond test session. Animals that failed to meet this predefined conditioning criterion were excluded from further analysis. This criterion was established to ensure that only animals showing evidence of successful conditioning were included in subsequent reinstatement and reconsolidation procedures. In Experiment 1, cocaine was replaced with NPCE, which was administered during the Cond phase to evaluate whether the extract itself could induce conditioned place preference or aversion. Following completion of the Pre-C and Cond phases, the experimental groups were established as described in Table 1.

#### 2.4.1. Experiment 1: Evaluation of NPCE in Acquisition of CPP

Experiment 1 was adapted from Viudez-Martínez et al. (2019) [3] and aimed to evaluate the intrinsic rewarding or aversive properties of NPCE. Animals (*n* = 20) underwent the Pre-C phase as described above. On day 4, animals were randomly assigned to two groups (*n* = 10) and received VEH (0.01 mL/g), followed by confinement in their initially preferred chamber for 15 min. On day 5, animals received either NPCE (equivalent to 20 mg/kg CBD content) or VEH and were confined to the initially non-preferred chamber for 15 min. Alternate-day conditioning continued until day 14. On day 15, animals were allowed to freely explore the CPPb for 15 min.

#### 2.4.2. Experiment 2: Evaluation of NPCE on the Reinstatement of CPP Induced by Cocaine

Experiment 2 was adapted from de Carvalho and Takahashi et al. [9] and aimed to evaluate the effects of NPCE on cocaine-induced CPP reinstatement. Following the Pre-C and Cond phases, animals (*n* = 20) that met the conditioning criterion remained in their home cages until day 14. On day 15, animals underwent a memory reactivation session consisting of confinement to the cocaine-paired chamber for 10 min, immediately followed by administration of NPCE (20 mg/kg, CBD-equivalent dose) or VEH. Animals subsequently remained in their home cages until day 33. On day 34, a post-reactivation CPP test was conducted by allowing animals to freely explore the CPP apparatus for 15 min in a drug-free state under continuous video recording. The extinction phase began on day 35 and lasted one week. During this period, animals were placed daily in the VEH-paired chamber for 20 min in the morning and, after a 6-h interval, in the drug-paired chamber for an additional 20 min. On day 43, extinction was considered achieved when no significant preference for the drug-paired chamber relative to Pre-C values was observed. On day 44, animals received a priming dose of cocaine (7.5 mg/kg, i.p.) and were tested for reinstatement. On day 51, animals were exposed to a 5-min auditory stressor consisting of continuous broadband white noise (100 dB, 500–8000 Hz), previously validated to induce acute stress responses and reinstatement-like behavior in rodent CPP paradigms [35]. Immediately afterward, animals were allowed to freely explore the CPP apparatus for 15 min.

#### 2.4.3. Experiment 3: Evaluation of NPCE in the Reconsolidation of CPP Induced by Cocaine

In Experiment 3, the memory reconsolidation procedure was adapted from the protocol described by de Carvalho et al. [9]. Following completion of the Pre-C and Cond phases, animals (*n* = 20) that met the conditioning criterion remained in their home cages until day 14. On day 15, animals were placed in the conditioned chamber for 10 min to induce memory reactivation, and immediately afterward, animals received either NPCE (20 mg/kg, CBD-equivalent dose) or VEH. Post-reactivation memory tests were conducted on days 22 and 29, during which animals were allowed to freely explore the CPPb for 15 min in a drug-free state.

### 2.5. Software and Statistical Analysis

Behavioral activity was recorded using digital video cameras (Brave 7 LE) and analyzed using AnyMaze^®^ software (version 7.15, Stoelting Co.). Statistical analyses were performed using GraphPad Prism (version 9.5.1, GraphPad Inc., Boston, MA, USA), with a significance level set at α = 0.05. Prior to analysis, data were assessed for normality using the Shapiro–Wilk test, complemented by additional normality tests (D’Agostino–Pearson, Anderson–Darling, and Kolmogorov–Smirnov) applied to the raw data within each group (Supplementary Material S3). Homogeneity of variances was assessed using Levene’s test for all between-group comparisons. For pooled comparisons between the pre-conditioning (Pre-C) and conditioning (Cond) phases, paired *t*-tests were used to confirm successful CPP acquisition. In Experiment 1, comparisons between the NPCE- and VEH-conditioned groups during the final CPP test were performed using unpaired two-tailed *t*-tests. For Experiments 2 and 3, which involved repeated assessments across experimental phases, data were analyzed using two-way repeated-measures ANOVA, with phase as the within-subject factor and treatment as the between-subject factor. When the assumption of sphericity was violated, degrees of freedom were adjusted using the Greenhouse–Geisser correction. Significant interactions or main effects were followed by Bonferroni-corrected post hoc comparisons to control for family-wise type I error. Results are presented as mean ± standard error of the mean (SEM). Exact *p*-values, test statistics, degrees of freedom, and effect sizes (Cohen’s d) with 95% confidence intervals are reported where appropriate.

### 2.6. Declaration of Generative AI and AI-Assisted Technologies in the Writing Process

During the preparation of this work, the authors used ChatGPT (OpenAI, GPT-4) to improve grammar, spelling, sentence structure, and language clarity. The authors subsequently reviewed and edited the generated text as needed and assume full responsibility for the final version of the manuscript.

## 3. Results

Of the 60 animals included in the study, 31 displayed an initial preference for the gray chamber during the Pre-C phase, whereas the remaining 29 preferred the blue chamber. Prior to conditioning, no baseline chamber preference was observed across experimental cohorts.

**Figure 1:**
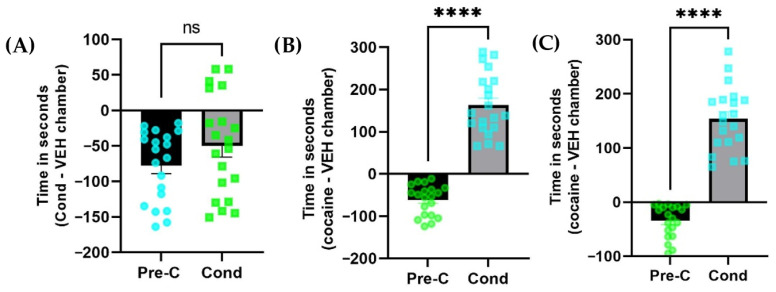
Pre-conditioning (Pre-C) and conditioning (Cond) results of pooled groups for (**A**) Experiment 1, (**B**) Experiment 2 and (**C**) Experiment 3 (*n* = 10 per group). Data are presented as means ± standard error of the mean (SEM). Each panel shows the comparison between Pre-C and Cond phases, where an increase in time spent in the conditioned chamber indicates successful CPP acquisition. (****) for *p* < 0.0001 versus Pre-C, ns, not significant.

Comparisons between the Pre-C and Cond phases for Experiments 1, 2, and 3 are presented in Figure 1. For all experiments, results are expressed as the difference in mean exploration time between the treatment-paired (conditioned) chamber and the vehicle-paired (VEH) chamber. In Experiment 1 (Figure 1A), which evaluated the intrinsic motivational properties of NPCE, no significant differences were observed between the Pre-C and Cond phases (paired *t*-test, t(19) = 0.29, *p* = 0.772, 95% CI [−49.17, 37.07]). In contrast, Experiments 2 and 3 (Figure 1B,C) showed a significant increase in the time spent in the conditioned chamber from the Pre-C to the Cond phase, confirming successful acquisition of cocaine-induced CPP prior to the subsequent intervention phases (Experiment 2: paired *t*-test, t(19) = 9.64, *p* < 0.0001, 95% CI [182.9, 284.3]; Experiment 3: paired *t*-test, t(19) = 12.09, *p* < 0.0001, 95% CI [159.0, 225.5]).

### 3.1. Experiment 1: Results of Evaluation of NPCE in the Acquisition of CPP

**Figure 2:**
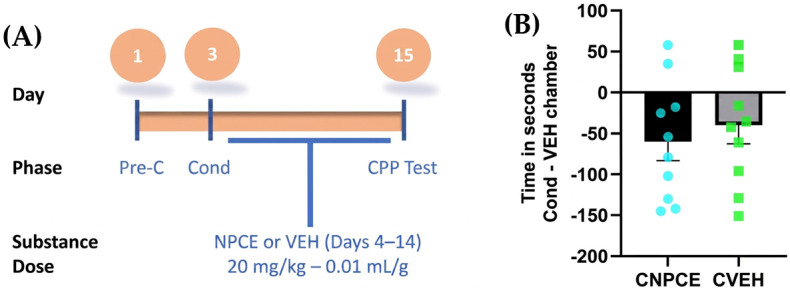
Effect of non-psychoactive cannabis extract (NPCE) on the acquisition of conditioned place preference (CPP). (**A**) Timeline of the behavioral procedure for Experiment 1. The paradigm consisted of a pre-conditioning phase (Pre-C), a conditioning phase (Cond), and a final test phase (CPP Test) on Day 15. During the conditioning phase (Days 4–14), animals were administered either NPCE (20 mg/kg, CBD-equivalent dose, approximately 48.7 mg/kg total extract) or vehicle (VEH; 0.01 mL/g). (**B**) Place preference scores during the CPP test, calculated as the difference in time spent between the drug-paired (conditioned) chamber and the vehicle-paired chamber (expressed in seconds). Data are presented as mean ± SEM (*n* = 10 per group), with scatter points representing individual animal scores. CNPCE: NPCE-conditioned group; CVEH: Vehicle-conditioned group.

In experiment 1, 20 mice were evaluated. Ten animals displayed an initial preference for the gray chamber, whereas the remaining ten preferred the blue chamber. To evaluate the intrinsic motivational properties of NPCE, comparisons between groups were conducted during the final test phase (Figure 2). Prior to parametric analyses, normality was assessed using the Shapiro–Wilk test, and homogeneity of variances was evaluated using Levene’s test for between-group comparisons. All datasets met the assumptions of normality and homoscedasticity (*p* > 0.05), supporting the use of parametric statistical tests.

To evaluate the intrinsic motivational properties of NPCE, comparisons between groups were conducted during the final test phase. No significant differences were observed between the NPCE- and VEH-conditioned groups (unpaired *t*-test, *t*(18) = 0.63, *p* = 0.536, Cohen’s d = 0.28, 95% CI [−47.18, 87.58]). These findings indicate that NPCE did not induce conditioned place preference or aversion under the conditions tested and are consistent with the absence of intrinsic reinforcing or aversive effects.

### 3.2. Experiment 2: Results of the Evaluation of NPCE in Cocaine-Induced CPP Reinstatement

**Figure 3:**
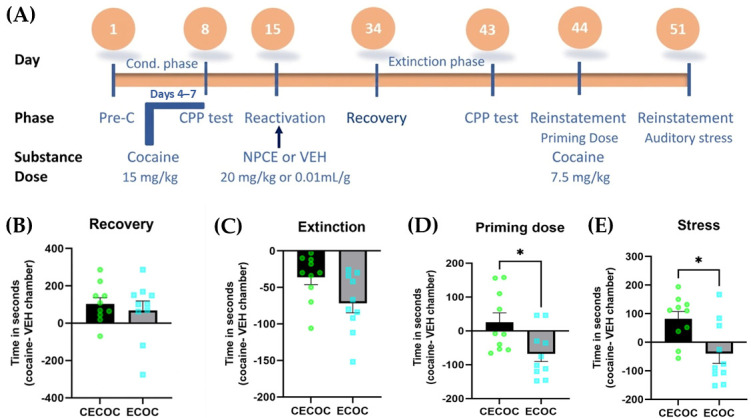
Effect of NPCE on extinction, and reinstatement of cocaine-induced CPP. (**A**) Timeline of the behavioral procedures for Experiment 2. The paradigm includes pre-conditioning (Pre-C), conditioning with cocaine (15 mg/kg), memory reactivation followed by the administration of either NPCE (20 mg/kg, CBD-equivalent dose, approximately 48.7 mg/kg total extract) or vehicle (VEH; 0.01 mL/g), an extinction phase, and reinstatement tests triggered by a cocaine priming dose (7.5 mg/kg) and auditory stress exposure. (**B**–**E**) Place preference scores, calculated as the difference in time spent between the cocaine-paired chamber and the vehicle-paired chamber (expressed in seconds). Data represent the (**B**) recovery phase, (**C**) extinction phase, (**D**) priming-induced reinstatement, and (**E**) stress-induced reinstatement. Data are presented as mean ± SEM (*n* = 10 per group), with *p* scatter points representing individual animal scores. CECOC: Control group (cocaine + vehicle); ECOC: Experimental group (cocaine + NPCE). * *p* < 0.05 indicates a statistically significant difference compared to the CECOC group.

Results of Experiment 2 are presented in Figure 3. Prior to parametric analyses, normality was assessed using the Shapiro–Wilk test, and homogeneity of variances was evaluated using Levene’s test. All datasets met the assumptions of normality and homoscedasticity (*p* > 0.05), supporting the use of parametric statistical tests. Because the assumption of sphericity was violated (Supplementary Material S3), degrees of freedom for the repeated-measures factor were adjusted using the Greenhouse–Geisser correction. To account for the repeated-measures structure of the CPP paradigm across experimental phases (Cond, extinction, and reinstatement), data were analyzed using a two-way repeated-measures ANOVA, with phase as the within-subject factor and treatment (CECOC [VEH, *n* = 10] vs. ECOC [NPCE 20 mg/kg, *n* = 10]) as the between-subject factor. The analysis revealed a significant main effect of phase (F(2.884, 51.91) = 21.84, *p* < 0.0001), a significant main effect of treatment (F(1,18) = 4.683, *p* = 0.0442), and a significant phase × treatment interaction (F(5,90) = 2.565, *p* = 0.0323). Post hoc comparisons using the Bonferoni correction revealed no significant differences between groups during the Pre-C, conditioning, recovery, or ex-tinction phases (*p* > 0.05). However, significant between-group differences emerged during reinstatement. NPCE-treated animals (ECOC) showed a significant reduction in preference compared to controls (CECOC) following both the priming dose (*p* = 0.0419) and stress exposure (*p* = 0.0201).

### 3.3. Experiment 3: Results of the Evaluation of NPCE in Cocaine-Induced CPP Reconsolidation

**Figure 4:**
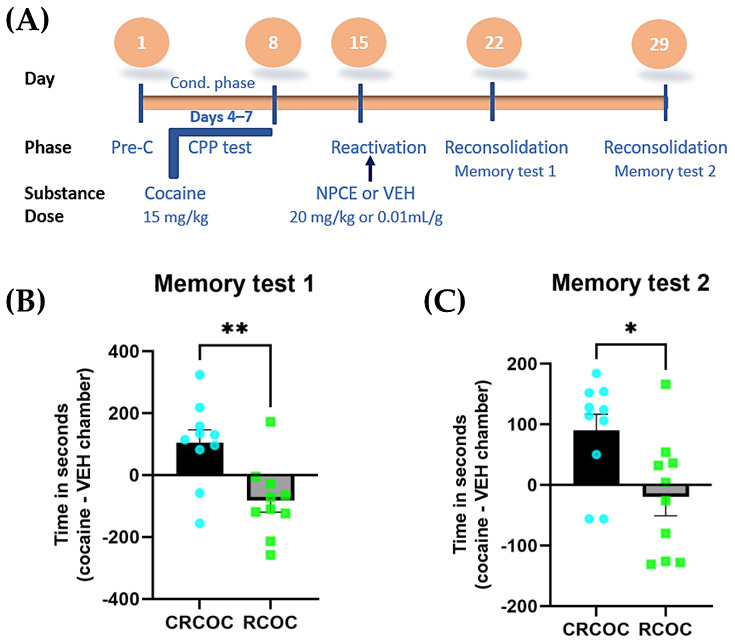
Effect of NPCE on the reconsolidation of cocaine-induced CPP memory. (**A**) Timeline of the behavioral procedures for Experiment 3. The paradigm includes pre-conditioning (Pre-C), condition with cocaine (15 mg/kg), memory reactivation followed by administration of NPCE (20 mg/kg, CBD-equivalent dose, approximately 48.7 mg/kg total extract) or vehicle (VEH; 0.01 mL/g), and two subsequent memory tests conducted at 7 days (Memory Test 1) and 14 days (Memory Test 2) post-reactivation. (**B**,**C**) Place preference scores, calculated as the difference in time spent between the cocaine-paired and vehicle-paired chambers (seconds), during (**B**) Memory Test 1 and (**C**) Memory Test 2. Data are presented as mean ± SEM (*n* = 10 per group), with scatter points representing individual animal scores. CRCOC: Control group (cocaine + vehicle); RCOC: NPCE-treated group (cocaine + NPCE). * *p* < 0.05 and ** *p*< 0.01 versus the CRCOC group.

Results of Experiment 3 are presented in Figure 4. Prior to parametric analyses, normality was assessed using the Shapiro–Wilk test, and homogeneity of variances was evaluated using Levene’s test. All datasets met the assumptions of normality and homoscedasticity (*p* > 0.05), supporting the use of parametric statistical tests. To account for the repeated-measures structure of the CPP paradigm across experimental phases, data were analyzed using a two-way repeated-measures ANOVA with Geisser–Greenhouse correction (Supplementary Material S3), with phase as the within-subject factor and treatment (CRCOC [VEH, *n* = 10] vs. RCOC [NPCE 20 mg/kg, *n* = 10]) as the between-subject factor. The analysis revealed a significant phase × treat-ment interaction (F(3, 54) = 8.261, *p* = 0.0001), indicating that the temporal evolution of preference scores differed between groups. Significant main effects of phase (F(2.225, 40.05) = 14.03, *p* < 0.0001) and treatment (F(1, 18) = 6.879, *p* = 0.0173) were also observed, with NPCE-treated animals exhibiting overall lower preference scores compared to controls.

Given the significant interaction, post hoc comparisons were performed using the Bonferroni correction. No significant differences between groups were observed during the Pre-C (mean difference = 2.70, 95% CI [−91.90 to 97.30], *p* > 0.9999) or conditioning phases (mean difference = −44.70, 95% CI [−137.2 to 47.84], *p* = 0.7544). In contrast, significant between-group differences emerged at both post-intervention time points, with NPCE-treated animals showing lower preference scores compared to controls during Test 1 (mean difference = 186.3, 95% CI [29.23 to 343.4], *p* = 0.0163) and Test 2 (mean difference = 128.1, 95% CI [19.32 to 236.9], *p* = 0.0176). Overall, these results indicate that NPCE administration following memory reactivation significantly disrupts the reconsolidation of cocaine-associated contextual memories and produces a sustained attenuation of conditioned preference over two weeks.

## 4. Discussion

This study evaluated the effects of an NPCE on cocaine-associated memory processes, including reinstatement and reconsolidation, using a cocaine-induced CPP model. Importantly, animals did not exhibit an initial preference for either chamber, supporting the validity of the CPP paradigm and indicating the absence of baseline contextual bias prior to conditioning.

In Experiment 1, NPCE (20 mg/kg, CBD-equivalent dose) did not induce conditioned preference or aversion, indicating a lack of intrinsic rewarding or aversive properties (Figure 2B). These findings are consistent with previous reports showing that CBD alone does not alter CPP acquisition or related CPP-related behavioral responses [3]. Notably, cannabinoid-induced behavioral effects are highly dependent on dose and phytochemical composition. Prior studies have shown that higher doses of THC or CBD, as well as cannabis extracts with elevated THC content, can produce measurable behavioral alterations [36]. Specifically, low doses of THC (below 0.5 mg/kg) have been associated with facilitation of extinction in cocaine-induced CPP [28], whereas higher doses may impair working memory and induce aversive responses to the conditioned context [37,38]. In this context, the use of a non-psychoactive extract characterized by a high CBD:THC ratio (58:1) and approximately 0.34 mg/kg THC likely contributed to the absence of motivational effects during acquisition.

Across Experiments 2 and 3, NPCE significantly attenuated both priming- and stress-induced reinstatement and interfered with reconsolidation-related processes associated with cocaine-conditioned memories. In contrast, no significant effects were observed during extinction or recovery phases, consistent with previous evidence indicating that CBD does not consistently modulate extinction learning [39]. Together, these findings support the hypothesis that cannabinoid-based interventions may preferentially target memory reactivation-dependent processes, such as reinstatement and reconsolidation, rather than extinction learning *per se*. However, these effects were observed within a preclinical CPP model and should not be directly extrapolated to clinical outcomes. Current evidence suggests that therapeutic potential of CBD in substance use disorders may arise, in part, from its capacity to disrupt drug-related memory reactivation and reconsolidation processes, as demonstrated in CPP paradigms [9,40]. In the present study, administration of NPCE immediately following memory reactivation produced sustained effects on cocaine-associated contextual memories. These findings suggest that NPCE may interfere with reactivation-dependent memory processes involved in the persistence and reinstatement of cocaine-associated behaviors [41]. The neurobiological mechanisms underlying these effects were not directly assessed in the present study. Nevertheless, previous literature provides plausible frameworks for interpreting the observed behavioral outcomes. For instance, reconsolidation of drug-associated memories has been linked to cannabinoid receptor type 1 (CB1) signaling [42,43,44,45] as well as dopaminergic modulation within the medial prefrontal cortex and associated limbic circuits [32,46]. Similarly, intracellular pathways such as cyclic adenosine monophosphate/protein kinase A (cAMP/PKA) signaling have been implicated in memory destabilization and reconsolidation processes [41,47]. These mechanisms remain hypothetical in the context of the present findings and should be interpreted cautiously.

Beyond memory reconsolidation, the attenuation of stress-induced reinstatement observed in this study may be related to the modulation of stress-responsive neurocircuitry. Stress-induced relapse is known to involve activation of corticotropin-releasing factor (CRF) and noradrenergic systems in regions such as the bed nucleus of the stria terminalis (BNST), hippocampus, and prefrontal cortex [48,49,50]. In addition, serotonergic signaling, particularly via the 5-HT_1A_ receptor, has been implicated in the regulation of stress responses and relapse vulnerability [51]. CBD, the major component of NPCE, has been shown to act as a partial agonist at 5-HT_1A_ receptors and to modulate drug-seeking behavior through interactions involving CB2 receptors, transient receptor potential vanilloid 1 (TRPV1), and dopaminergic systems [52,53]. These mechanisms may provide a plausible neurobiological context for the reduced reinstatement observed; however, they were not directly evaluated in the present study.

Importantly, cannabinoid effects on stress-related systems are complex and context-dependent [54]. Evidence from both human and animal studies indicates that acute cannabinoid exposure can increase stress hormone levels [55], whereas chronic exposure has been associated with blunted stress reactivity and alterations in cortisol dynamics, including a flattened cortisol awakening response [56]. These findings suggest that cannabinoid exposure may not uniformly normalize hypothalamic–pituitary–adrenal (HPA) axis function but rather induce adaptive or maladaptive neuroendocrine changes depending on dose, duration, and population characteristics. Accordingly, the relationship between cannabinoids and HPA axis regulation appears to be bidirectional and population-dependent, warranting caution when interpreting the potential protective effects of NPCE.

### Limitations and Future Directions

These findings should be interpreted in light of methodological limitations. These include the exclusive use of male CD1 mice, the evaluation of a single NPCE dose and administration route, and the absence of blinding during data analysis. Although behavioral outcomes were obtained through automated AnyMaze^®^ tracking without manual scoring, the lack of prospective blinding remains a potential source of bias. To improve transparency, the original AnyMaze^®^ raw output files with anonymized group labels are provided in Supplementary Material S2. Male mice were used to maintain consistency with previous studies employing similar CPP paradigms and CBD interventions [7,10,15], although this limits the generalizability of the findings. Sex differences have been widely reported in cocaine-related behaviors, including reinforcement, extinction, and reinstatement [57,58], as well as in hypothalamic–pituitary–adrenal (HPA) axis reactivity to stress [54,59]. Furthermore, cannabinoid pharmacology, including the behavioral and neurobiological effects of CBD, may differ between males and females [60]. Future studies incorporating both sexes will be necessary to determine whether the effects of NPCE are sex-dependent.

Another limitation of this study is the absence of a CBD-only control group to directly compare the effects of NPCE on CPP-related outcomes. Nevertheless, several studies in the scientific literature provide a useful framework for comparison with the present findings [7,10,15]. The NPCE used in this study is a complex botanical formulation containing multiple cannabinoids and terpenes (Supplementary Material S1), which may individually or collectively contribute to the observed effects. However, in the absence of isolated control conditions, the specific contribution of CBD, minor cannabinoids, or terpenes cannot be determined. Although interactions among these constituents have been proposed in the literature, including the concept of the “entourage effect”, the present experimental design does not allow us to empirically attribute the observed effects to synergistic mechanisms rather than to the action of CBD alone. Therefore, interpretations regarding such interactions should be considered speculative. Future studies incorporating isolated compounds and controlled combinations will be necessary to clarify these contributions.

Additionally, the present study evaluated only a single NPCE dose and administration route, thereby precluding dose–response analyses and limiting conclusions regarding the optimal pharmacological profile of the extract. Furthermore, the behavioral assessment relied exclusively on the CPP paradigm, which, although widely established for investigating reward-associated learning and contextual memory processes, captures only specific dimensions of addiction-related behavior. We acknowledge the absence of a dedicated locomotor activity assessment as a limitation of the present study, and future research incorporating specific experimental designs to evaluate this parameter is warranted. The incorporation of complementary models, particularly cocaine self-administration paradigms, would strengthen the translational relevance of these findings and provide a more comprehensive characterization of the therapeutic potential of NPCE.

## 5. Conclusions

In conclusion, the present study provides evidence that NPCE attenuates priming- and stress-induced reinstatement and produces effects consistent with disruption of reconsolidation-like processes in cocaine-induced CPP in mice, without producing reinforcing or aversive effects during acquisition. These findings support the hypothesis that a low-THC CBD-rich cannabis extract may modulate drug-associated memory processes and relapse-like behaviors in preclinical models. While these results support further investigation of NPCE as a candidate intervention targeting maladaptive reward memories, additional studies are required to confirm these effects across other behavioral paradigms and experimental conditions, as well as to elucidate the underlying neurobiological mechanisms.

## Figures and Tables

**Table 1:** Experimental groups and treatments. Overview of experimental groups, conditioning procedures, and post-reactivation treatments.

CPP Study	Group Label	Substance Administered During Conditioning/Dose	Post-Reactivation Treatment/Dose
Experiment 1. NPCE-induced CPP (acquisition)	CNPCE (NPCE-conditioned group, *n* = 10)	NPCE (20 mg/kg, CBD-equivalent dose, approximately 48.7 mg/kg total extract)	-
	CVEH (vehicle-conditioned group, *n* = 10)	VEH (0.01 mL/g)	-
Experiment 2. Reinstatement (priming- and stress-induced)	ECOC (NPCE-treated group, *n* = 10)	Cocaine 15 mg/kg	NPCE (20 mg/kg, CBD-equivalent dose, approximately 48.7 mg/kg total extract)
	CECOC (vehicle-treated control group, *n* = 10)		VEH (0.01 mL/g)
Experiment 3. Reconsolidation	RCOC (NPCE-treated reconsolidation group, *n* = 10)		NPCE (20 mg/kg, CBD-equivalent dose, approximately 48.7 mg/kg total extract)
	CRCOC (vehicle-treated reconsolidation control group, *n* = 10)		VEH (0.01 mL/g)

Vehicle solution (VEH), cocaine (COC), low-THC CBD-rich cannabis extract (NPCE). A total of 60 animals were planned for the study to complete the six experimental groups (*n* = 10/group). During the Pre-C and conditioning phases, three animals met exclusion criteria: one due to strong initial chamber preference (>10 min in one chamber) and two due to failure to meet the conditioning criterion during the test session. These animals were excluded before final group allocation and were immediately replaced while group distribution was still ongoing, resulting in a final sample size of *n* = 10 per group for all statistical analyses.

## Data Availability

The raw behavioral datasets generated and analyzed during the current study are provided as Supplementary Material S2 associated with this manuscript. In addition, the complete statistical analysis data file corresponding to the analyses reported in the manuscript is available as Supplementary Material S3. Further information or derived datasets supporting the findings of this study may be obtained from the corresponding author upon reasonable request, in accordance with institutional policies governing data management and ethical considerations related to animal research.

## Supplementary Materials

The following supporting information can be downloaded at: https://www.mdpi.com/article/10.3390/brainsci16060585/s1, Supplementary Material S1. Detailed Chromatograms and Chemical Composition of NPCE. Supplementary Materials S2. Raw Behavioral Data. Supplementary Materials S3. Detailed statistical analysis outputs for all experiments.
