# Supplementary material for: Non-Psychoactive Cannabis Extract Disrupts Reinstatement and Reconsolidation in Cocaine-Induced Conditioned Place Preference in Mice
Source: Brain Sci. 2026 May 29;16(6):585. doi: 10.3390/brainsci16060585 (PMC13297324; doi:10.3390/brainsci16060585)
Supplement: Supplementary file 1 [file brainsci-16-00585-s001.zip › Supplementary Material S3.pdf]

SUPPLEMENTARY MATERIAL S3

NON-PSYCHOACTIVE CANNABIS EXTRACT DISRUPTS REINSTATEMENT AND RECONSOLIDATION IN COCAINE-INDUCED CONDITIONED PLACE PREFERENCE IN MICE

Supplementary Material S3. Detailed statistical analysis outputs for all experiments.

Normality was assessed for each dataset using multiple complementary tests, including D'Agostino–Pearson, Anderson–Darling, Shapiro–Wilk, and Kolmogorov–Smirnov tests. Across all groups (n = 10 per group) of Experiment 2, none of the datasets showed significant deviations from normality. The D'Agostino–Pearson test yielded non-significant results across all comparisons (K^2 values ranging from 0.2313 to 2.964; $p = 0.2271$–0.8908). Similarly, the Anderson–Darling test indicated no violations of normality (A^2 values ranging from 0.1865 to 0.6605; $p = 0.0587$–0.8737). The Shapiro–Wilk test confirmed normal distribution in all groups (W = 0.8500–0.9800; $p = 0.0581$–0.9650), and the Kolmogorov–Smirnov test further supported these findings (KS distance = 0.1396–0.2463; $p > 0.0866$ in all cases). Overall, all datasets met the assumption of normality ($p > 0.05$), supporting the use of parametric statistical analyses. For Experiment 3, normality was assessed for each dataset using multiple complementary tests, including D'Agostino–Pearson, Anderson–Darling, Shapiro–Wilk, and Kolmogorov–Smirnov tests. Across all conditions (n = 10 per group), none of the datasets showed significant deviations from normality. The D'Agostino–Pearson test yielded non-significant results across all comparisons (K^2 values ranging from 0.3721 to 3.032; $p = 0.2195$–0.8302). Similarly, the Anderson–Darling test indicated no violations of normality (A^2 values ranging from 0.2493 to 0.6492; $p = 0.0630$–0.6657). The Shapiro–Wilk test confirmed normal distribution in all groups (W = 0.8640–0.9666; $p = 0.0850$–0.8576), and the Kolmogorov–Smirnov test further supported these findings (KS distance = 0.1623–0.2205; all $p > 0.1000$). Overall, all datasets met the assumption of normality ($p > 0.05$), supporting the use of parametric statistical analyses.

Table S1. Summary of normality, homogeneity of variance, and sphericity assumption checks across all experiments and statistical models

**Experiment**	**Normality (Shapiro–Wilk on residuals)**	**Homogeneity of variances (Levene's test)**	**Sphericity**	**Correction applied**
Experiment 1 (CPP intrinsic motivation)	$p > 0.05$ (all groups) ✓	$p > 0.05$ ✓	Not applicable	None (unpaired t-test)
Experiment 2 (Cocaine reinstatement)	$p > 0.05$ (all groups) ✓	$p > 0.05$ ✓	Violated ✗ (Time)	Greenhouse–Geisser correction applied

Experiment 3 (Cocaine reconsolidation)	$p > 0.05$ (all groups) ✓	$p > 0.05$ ✓	Violated ✗ (Time, ε = 0.7417)	Greenhouse–Geisser correction applied

Note: ✓ indicates that the statistical assumption was met; ✗ indicates violation of the statistical assumption. ε represents the Greenhouse–Geisser epsilon value used for sphericity correction. CPP: conditioned place preference.

Table S2. Two-way repeated-measures ANOVA results for cocaine reinstatement for Experiment 2.

Source of Variation	% of total variation	F (DFn, DFd)	P value	P value summary	Significant?	Geisser–Greenhouse ε
Time × Column Factor	6.162	F(5, 90) = 3.089	0.0128	*	Yes	—
Time	45.36	F(2.767, 49.81) = 22.74	<0.0001	****	Yes	0.5535
Column Factor	3.071	F(1, 18) = 5.822	0.0267	*	Yes	—
Subject	9.494	F(18, 90) = 1.322	0.1936	ns	No	—

Note: * p < 0.05; **** p < 0.0001; ns, not significant. DFn: degrees of freedom numerator; DFd: degrees of freedom denominator; ε: Greenhouse–Geisser epsilon correction factor.

Table S3. Detailed two-way repeated-measures ANOVA statistics for Experiment 2 (cocaine-induced reinstatement).

Effect	SS	DF	MS	F (DFn, DFd)	P value
Time × Column Factor	107885	5	21577	F(5, 90) = 3.089	0.0128
Time	794219	2.767	158844	F(2.767, 49.81) = 22.74	<0.0001
Column Factor	53763	1	53763	F(1, 18) = 5.822	0.0267
Subject	166222	18	9235	F(18, 90) = 1.322	0.1936
Residual	628651	90	6985	—	—

Note: SS, sum of squares; DF, degrees of freedom; MS, mean square; DFn, degrees of freedom numerator; DFd, degrees of freedom denominator. ε represents the Greenhouse–Geisser epsilon correction factor applied when sphericity assumptions were violated.

Table S4. Group comparison statistics for ECOC and CECOC during cocaine-induced reinstatement in Experiment 2.

Parameter	Value
Mean ECOC	-0.7667
Mean CECOC	41.57
Difference between means	-42.33
SE of difference	17.54
95% CI	-79.19 to -5.473

Note: SE, standard error; CI, confidence interval; ECOC, reinstatement group treated with cocaine; CECOC, reinstatement group treated with vehicle (VEH).

Table S5. Bonferroni-corrected post hoc comparisons between ECOC and CECOC across reinstatement phases (Experiment 2).

Phase	Mean Difference (ECOC – CECOC)	95% CI	Adjusted P value	Significance
Pre-C	44.00	-71.27 to 159.3	>0.9999	ns
Cond	6.60	-80.13 to 93.33	>0.9999	ns
Recovery	-34.40	-215.2 to 146.4	>0.9999	ns
Extinction	-35.60	-84.19 to 12.99	0.2582	ns
Priming Dose	-103.5	-204.2 to - 2.776	0.0419	*
Stress	-131.1	-246.0 to - 16.22	0.0201	*

Note: ECOC, reinstatement group treated with NPCE at 20 mg/kg; CECOC, reinstatement group treated with vehicle (VEH); CI, confidence interval; ns, not significant; * $p < 0.05$. Pre-C, pre-conditioning phase.

Table S6. Descriptive and comparative statistics across experimental phases for ECOC and CECOC groups in Experiment 2 (cocaine-induced reinstatement).

Phase	Mean ECOC	Mean CECOC	Mean Diff	SE	t	DF	N1	N2
Pre-C	-50.00	-94.00	44.00	38.72	1.136	17.33	10	10
Cond	164.9	158.3	6.60	29.05	0.2272	16.95	10	10
Recovery	69.10	103.5	-34.40	59.78	0.5754	15.42	10	10
Extinction	-71.90	-36.30	-35.60	16.28	2.187	16.97	10	10
Priming Dose	-67.80	35.70	-103.5	33.96	3.048	17.83	10	10
Stress	-48.90	82.20	-131.1	38.68	3.389	17.65	10	10

Note: ECOC, reinstatement group treated with NPCE at 20 mg/kg; CECOC, reinstatement group treated with vehicle (VEH); Mean Diff, mean difference; SE, standard error; DF, degrees of freedom; N1 and N2 represent the number of animals per group. Pre-C, pre-conditioning phase.

For repeated-measures analyses, sphericity was evaluated and Greenhouse–Geisser correction was applied when necessary. In addition, Q-Q plots of model residuals are provided to visually support the assumption of normality in the statistical models.

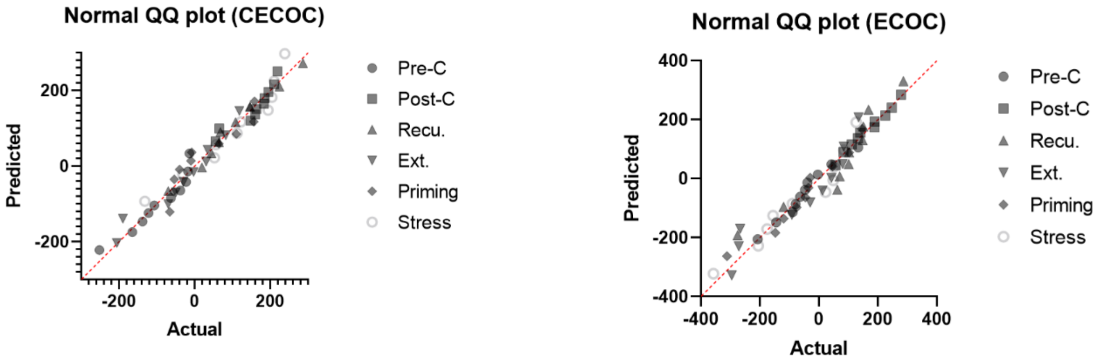

Figure S1. Q-Q plot of model residuals for the two-way repeated-measures ANOVA (Reinstatement). The plot illustrates the relationship between observed ("Actual") and predicted values derived from the model across all experimental phases (Pre-C, Post-C, Recovery, Extinction, Reinstatement 1, and Reinstatement 2). Data points from all phases are distributed along the reference diagonal line (red dashed line), indicating that residuals are approximately normally distributed and consistent with the assumption of normality required for parametric analyses.

Table S7. Two-way repeated-measures ANOVA results for cocaine reconsolidation experiment (Experiment 3).

Source of Variation	% of total variation	F (DFn, DFd)	P value	P value summary	Significant?	Geisser–Greenhouse ε
Time × Column Factor	14.67	F(3, 54) = 8.261	0.0001	***	Yes	—
Time	24.91	F(2.225, 40.05) = 14.03	<0.0001	****	Yes	0.7417
Column Factor	7.870	F(1, 18) = 6.879	0.0173	*	Yes	—
Subject	20.59	F(18, 54) = 1.933	0.0323	*	Yes	—

Note: * p < 0.05; *** p < 0.001; **** p < 0.0001. DFn, degrees of freedom numerator; DFd, degrees of freedom denominator; ε, Greenhouse–Geisser epsilon correction factor.

Table S8. Analysis of variance components for the two-way repeated-measures ANOVA in Experiment 3 (cocaine-induced reconsolidation).

Effect	SS	DF	MS	F (DFn, DFd)	P value
Time × Column Factor	172861	3	57620	F(3, 54) = 8.261	0.0001
Time	293604	2.225	97868	F(2.225, 40.05) = 14.03	<0.0001
Column Factor	92752	1	92752	F(1, 18) = 6.879	0.0173
Subject	242708	18	13484	F(18, 54) = 1.933	0.0323
Residual	376635	54	6975	—	—

Note: SS, sum of squares; DF, degrees of freedom; MS, mean square; DFn, degrees of freedom numerator; DFd, degrees of freedom denominator. ε represents the Greenhouse–Geisser epsilon correction factor applied when sphericity assumptions were violated.

Table S9. Summary statistics and mean differences between CRCOC and RCOC groups in Experiment 3 (cocaine-induced reconsolidation).

Parameter	Value
Mean CRCOC (VEH)	81.90
Mean RCOC (NPCE 20 mg/kg)	13.80
Difference between means	68.10
SE of difference	25.97
95% CI	13.55 to 122.7

Note: CRCOC (VEH), reconsolidation group treated with vehicle (VEH); RCOC (NPCE 20 mg/kg), reconsolidation group treated with NPCE at 20 mg/kg; SE, standard error; CI, confidence interval.

Table S10. Bonferroni-corrected post hoc comparisons between CRCOC (VEH) and RCOC (NPCE 20 mg/kg) across reconsolidation phases

Phase	Mean Difference (CRCOC – RCOC)	95% CI	Adjusted P value	Significance
Pre-C	2.70	-91.90 to 97.30	>0.9999	ns
Cond	-44.70	-137.2 to 47.84	0.7544	ns
TEST 1	186.3	29.23 to 343.4	0.0163	*
TEST 2	128.1	19.32 to 236.9	0.0176	*

Note: CRCOC, reconsolidation group treated with vehicle (VEH); RCOC, reconsolidation group treated with NPCE at 20 mg/kg; CI, confidence interval; ns, not significant; * $p < 0.05$; Pre-C, pre-conditioning phase.

Table S11. Group means and pairwise comparison statistics across experimental phases for CRCOC and RCOC groups in Experiment 3 (cocaine-induced reconsolidation).

Phase	Mean CRCOC (VEH)	Mean RCOC (NPCE 20 mg/kg)	Mean Diff	SE	t	DF	N1	N2
Pre-C	-4.600	-7.300	2.70	33.86	0.07974	16.93	10	10
Cond	127.0	171.7	-44.70	32.36	1.381	14.14	10	10
TEST 1	104.0	-82.30	186.3	56.52	3.296	17.73	10	10
TEST 2	101.2	-26.90	128.1	38.65	3.314	15.94	10	10

Note: CRCOC (VEH), reconsolidation group treated with vehicle (VEH); RCOC (NPCE 20 mg/kg), reconsolidation group treated with NPCE at 20 mg/kg; Mean Diff, mean difference; SE, standard error; DF, degrees of freedom; N1 and N2 represent the number of animals per group; Pre-C, pre-conditioning phase.

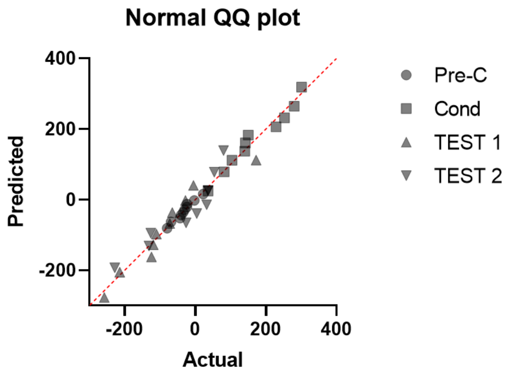

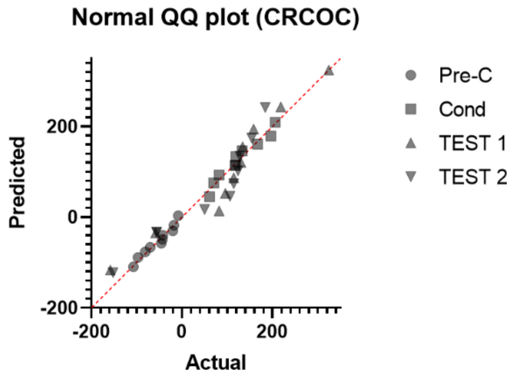

Figure S2. Q-Q plot of model residuals for the two-way repeated-measures ANOVA (Reconsolidation). Normal Q-Q plot of the model residuals obtained from the ANOVA analyses across experimental phases (Pre-C, Cond, TEST 1, and TEST 2). The observed values are plotted against the theoretical normal distribution. The red dashed line represents the expected distribution under normality. The distribution of residuals closely follows the diagonal line, indicating no substantial deviations from normality and supporting the validity of the parametric model assumptions.
