# Supplementary material for: Non-Psychoactive Cannabis Extract Disrupts Reinstatement and Reconsolidation in Cocaine-Induced Conditioned Place Preference in Mice
Source: Brain Sci. 2026 May 29;16(6):585. doi: 10.3390/brainsci16060585 (PMC13297324; doi:10.3390/brainsci16060585)
Supplement: Supplementary file 1 [file brainsci-16-00585-s001.zip › Supplementary Material S1.pdf]

SUPPLEMENTARY MATERIAL S1

NON-PSYCHOACTIVE CANNABIS EXTRACT DISRUPTS REINSTATEMENT AND RECONSOLIDATION IN COCAINE-INDUCED CONDITIONED PLACE PREFERENCE IN MICE

Supplementary Material S1. Detailed Chromatograms and Chemical Composition of NPCE

Chromatogram (TIC) of cannabinoids found in NPCE

Injection Details

Injection Name:	**NPCE**	*Run Time (min):*	**26.60**
Vial Number:	**122**	*Injection Volume:*	**1.00**
Injection Type:	**Unknown**	*Channel:*	**TIC**
Calibration Level:		*Wavelength:*	**n.a.**
Instrument Method:	**SPV_TORRE AL1310**	*Bandwidth:*	**n.a.**
Processing Method:	**CANNABIS**	*Dilution Factor:*	**1.0000**
Injection Date/Time:	**21/ene./26 17:30**	*Sample Weight:*	**1.0000**

Chromatogram

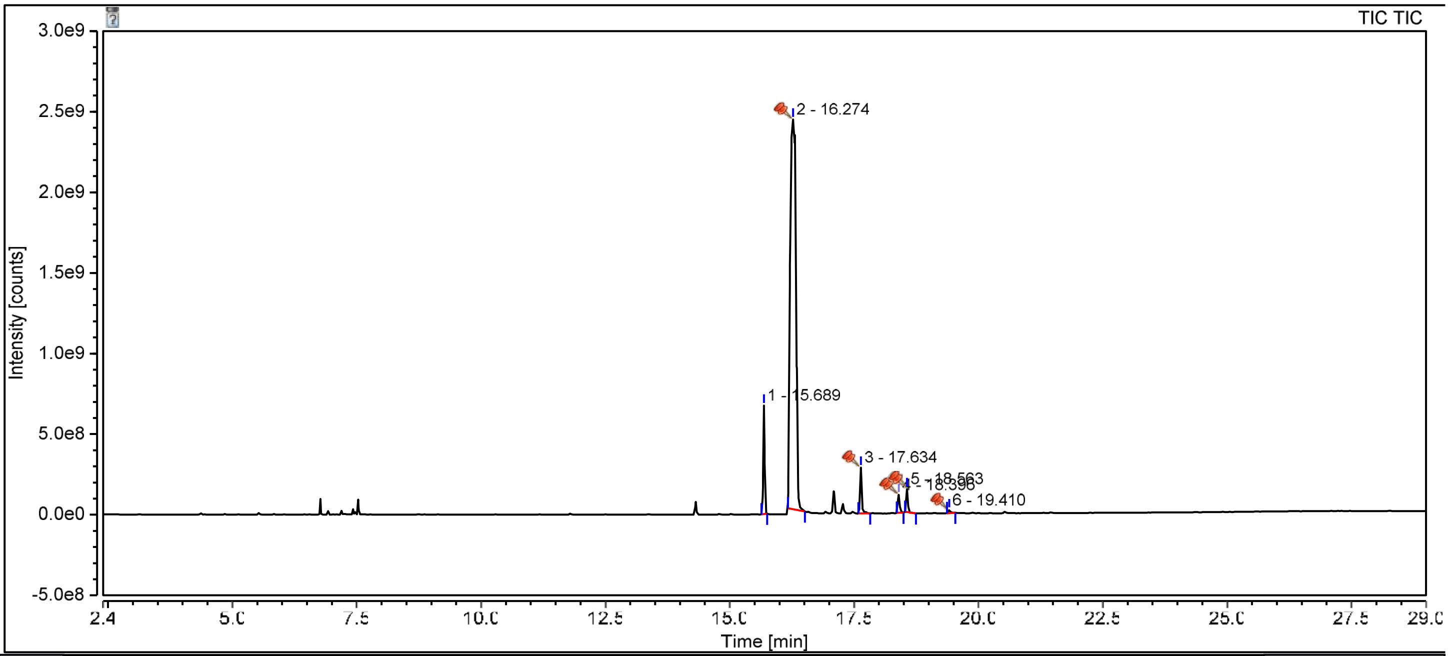

Integration Results

No.	Peak Name	Retention Time min	Area counts*min	Height counts	Rel.Area (GROUP) %	Resolution (EP)	Asymmetry (EP)
1	Tetracosano	15.689	21641334.142	672301483.201	0.00	4.18	0.94
2	CBD	16.274	335514661.400	2417297026.986	93.97	9.47	1.03
3	THC	17.634	11332157.802	283264931.718	3.17	13.25	1.24
4	CBG	18.396	4184601.162	111113078.030	1.17	2.86	1.31
5	CBN	18.563	5138588.086	141676007.886	1.44	12.21	1.11
6	CBC	19.410	874758.942	18173852.946	0.24	n.a.	1.24
**Total:**			**378686101.535**	**3643826380.767**	**100.00**	**41.96**	

Chromatogram (TIC) of terpenes found in NPCE

Injection Details

Injection Name:	**Terpenos NPCE 2**	*Run Time (min):*	**51.80**
Vial Number:	**42**	*Injection Volume:*	**1.00**
Injection Type:	**Unknown**	*Channel:*	**TIC**
Calibration Level:		*Wavelength:*	**n.a.**
Instrument Method:	**Solventes_SPME_Manual**	*Bandwidth:*	**n.a.**
Processing Method:	**Quantitative**	*Dilution Factor:*	**1.0000**
Injection Date/Time:	**02/feb./25 11:07**	*Sample Weight:*	**1.0000**

Chromatogram

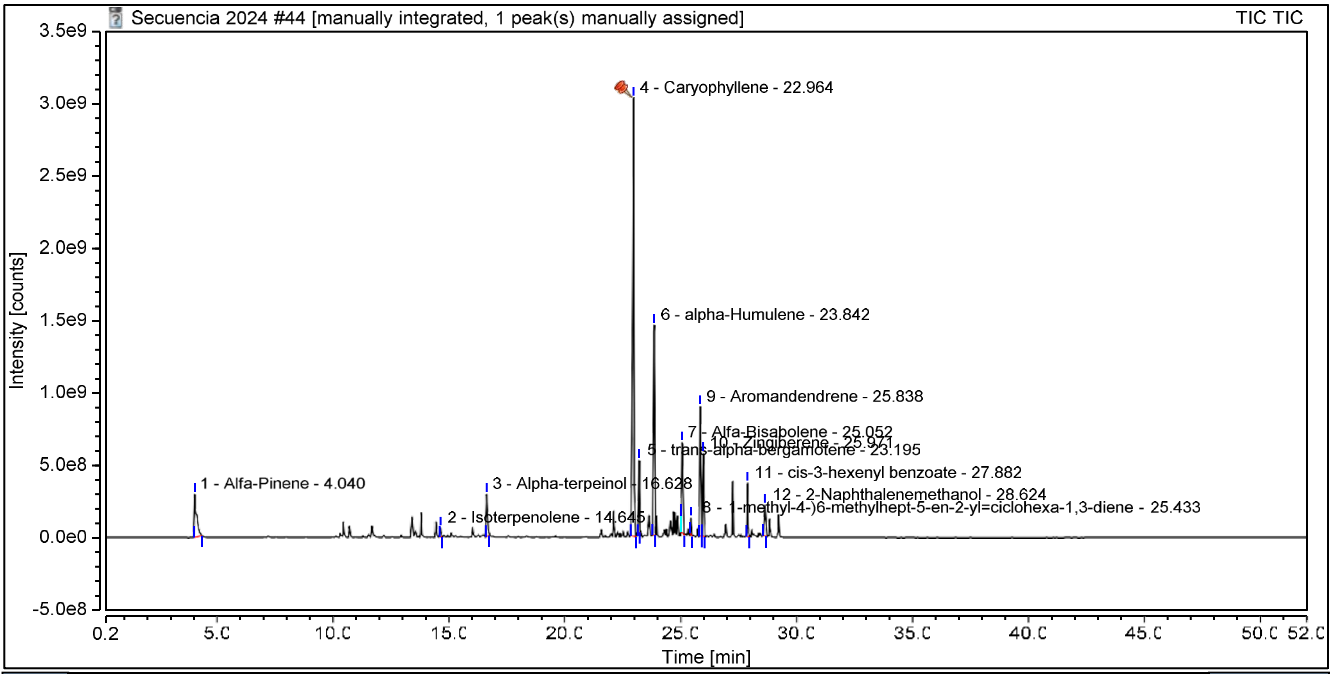

Integration Results

No.	Peak Name	Retention Time<br>min	Area<br>counts*min	Height<br>counts	Relative Area<br>%	Relative Height<br>%
1	Alfa-Pinene	4.040	29968583.966	297127167.279	6.70	3.53
2	Isoterpenolene	14.645	2831396.408	60148259.058	0.63	0.72
3	Alpha-terpeinol	16.628	15616400.938	289585138.973	3.49	3.44
4	Caryophyllene	22.964	200865686.684	3028602550.379	44.89	36.01
5	trans-alpha-bergamotene	23.195	18666248.578	505402325.299	4.17	6.01
6	alpha-Humulene	23.842	62929998.158	1450755296.696	14.06	17.25
7	Alfa-Bisabolene	25.052	24763000.777	621540323.883	5.53	7.39
8	1-methyl-4-)6-methylhept-	25.433	4455316.592	113145254.746	1.00	1.35
9	Aromandendrene	25.838	38780722.604	892806558.881	8.67	10.61
10	Zingiberene	25.971	22608336.732	572490521.655	5.05	6.81
11	cis-3-hexenyl benzoate	27.882	14546949.724	367823100.123	3.25	4.37
12	2-Naphthalenemethanol	28.624	11463049.117	211822718.808	2.56	2.52
**Total:**			**447495690.278**	**8411249215.780**	**100.00**	**100.00**
