# Supplementary material for: Non-Psychoactive Cannabis Extract Disrupts Reinstatement and Reconsolidation in Cocaine-Induced Conditioned Place Preference in Mice
Source: Brain Sci. 2026 May 29;16(6):585. doi: 10.3390/brainsci16060585 (PMC13297324; doi:10.3390/brainsci16060585)
Supplement: Supplementary file 1 [file brainsci-16-00585-s001.zip › Supplementary Material S2.pdf]

SUPPLEMENTARY MATERIAL S2

NON-PSYCHOACTIVE CANNABIS EXTRACT DISRUPTS REINSTATEMENT AND RECONSOLIDATION IN COCAINE-INDUCED CONDITIONED PLACE PREFERENCE IN MICE

Supplementary Material S2: Raw Behavioral Data

This document contains the raw behavioral datasets corresponding to Experiments 1, 2, and 3, which evaluated the effects of NPCE (20 mg/kg CBD-equivalent dose) on cocaine-induced conditioned place preference (CPP) and related relapse-like behavioral outcomes in mice.

For Experiments 1, 2, and 3, the dataset includes the raw CPP scores obtained across the different experimental phases.

Raw CPP Score Data Across Experimental Phases

The following tables present the raw individual CPP scores for all animals included in Experiments 1, 2, and 3. CPP scores were calculated as the difference in time spent between the drug-paired and vehicle-paired compartments.

Table S1. Time spent in compartments across experimental phases in Experiment 1.

Animal ID	Pre-C (s)	Cond (s)
1	-74	-158
2	-55	-41
3	-164	-92
4	-109	135
5	28	-31
6	-142	-118
7	-45	-67
8	-29	-22
9	-18	-143
10	-48	-38
11	-61	-145
12	-42	58
13	-151	-79
14	-96	-142
15	41	-18
16	-129	-102
17	58	-54
18	-163	35
19	1	-130
20	-35	-25

Table S2. Time spent in compartments across experimental phases in Experiment 2.

Animal ID	Pre-C (s)	Cond (s)	Recovery (s)	Extinction (s)	Priming Dose (s)	Stress (s)
1	-145	111	102	-84	-102	-76
2	-39	225	70	-34	46	107
3	-89	56	62	-152	-148	-106
4	42	247	150	-80	-76	64
5	-4	189	-276	-92	-146	-148
6	-64	132	148	-30	46	-152
7	133	83	-120	-26	-120	48
8	-79	188	101	-67	-30	-26
9	-47	140	168	-112	-36	-90
10	-208	278	286	-42	-112	-110
11	-38	195	286	-10	64	150
12	-138	160	146	-34	-40	38
13	-63	65	64	-70	-36	132
14	-252	55	44	-50	156	110
15	-165	210	146	-12	110	122
16	-18	185	224	-30	-10	-56
17	-23	148	-70	-18	-8	52
18	-107	219	108	-3	-3	112
19	-14	163	19	-30	-34	194
20	-122	183	68	-106	158	-32

Table S3. Time spent in compartments across experimental phases in Experiment 3.

Animal ID	Pre-C (s)	Cond (s)	Test 1 (s)	Test 2 (s)
1	-42	61	96	50
2	-8	167	130	184
3	46	206	218	56
4	20	133	114	154
5	-18	82	82	-56
6	70	119	134	128
7	-97	196	158	152
8	-81	118	-158	106
9	-43	118	-58	114
10	107	70	324	124
11	23	229	-5	-228
12	30	301	172	32

13	34	36	-119	-131
14	34	253	-28	80
15	40	280	-258	54
16	43	82	-65	-26
17	69	140	-72	4
18	-24	150	-214	36
19	-180	104	-124	-126
20	-142	142	-110	36

Note: Values represent CPP scores expressed as the difference in time spent between the cocaine-paired and non-paired compartments during each experimental phase.
